# Supplementary material for: Challenges in managing HIV and non-communicable diseases and health workers’ perception regarding integrated management of non-communicable diseases during routine HIV care in South Central Uganda: A qualitative study
Source: PLoS One. 2024 Aug 20;19(8):e0302290. doi: 10.1371/journal.pone.0302290 (PMC11335126; doi:10.1371/journal.pone.0302290)
Supplement: S1 File — https://doi.org/10.6084/m9.figshare.25551813. (DOCX) [file pone.0302290.s001.docx]

S1 File: Inclusivity in Global Research

<https://doi.org/10.6084/m9.figshare.25551813>
