# Supplementary material for: Challenges in managing HIV and non-communicable diseases and health workers’ perception regarding integrated management of non-communicable diseases during routine HIV care in South Central Uganda: A qualitative study
Source: PLoS One. 2024 Aug 20;19(8):e0302290. doi: 10.1371/journal.pone.0302290 (PMC11335126; doi:10.1371/journal.pone.0302290)
Supplement: S1 Checklist — https://doi.org/10.6084/m9.figshare.25262521.v1. (DOCX) [file pone.0302290.s002.docx]

**S1 Checklist 1 : Minimum dataset.**

<https://doi.org/10.6084/m9.figshare.25262521.v1>
