# Supplementary material for: Challenges in managing HIV and non-communicable diseases and health workers’ perception regarding integrated management of non-communicable diseases during routine HIV care in South Central Uganda: A qualitative study
Source: PLoS One. 2024 Aug 20;19(8):e0302290. doi: 10.1371/journal.pone.0302290 (PMC11335126; doi:10.1371/journal.pone.0302290)
Supplement: Attachment — Submitted filename: PONE-D-23-38985_JF.pdf [file pone.0302290.s003.pdf]

PLOS ONE

Challenges in managing Persons living with HIV with non-communicable diseases and the perception of health workers towards integrated management of non-communicable diseases during routine HIV care in South Central Uganda: A qualitative study.

--Manuscript Draft--

Manuscript Number:	PONE-D-23-38985
Article Type:	Research Article
Full Title:	Challenges in managing Persons living with HIV with non-communicable diseases and the perception of health workers towards integrated management of non-communicable diseases during routine HIV care in South Central Uganda: A qualitative study.
Short Title:	Perception of health workers towards integrated management of noncommunicable diseases during routine HIV care
Corresponding Author:	Asani Kasango, MPH<br>Rakai Health Sciences Program<br>Masaka, UGANDA
Keywords:	HIV, noncommunicable diseases, integrated management, health workers
Abstract:	Background<br><br>Non-communicable diseases have been reported to be common among adults living with HIV. However, data on health systems challenges in managing non-communicable diseases and the perception of health workers towards integrated management of non-communicable diseases in routine care are limited in rural Ugandan settings.<br>This study therefore explored health system challenges in managing PLHIV with non-communicable diseases and health workers' perception regarding integration of non-communicable diseases in routine HIV care in South Central Uganda.<br><br>Methods<br><br>In this qualitative study, we collected data from 20 purposively selected key informants from Kalisizo and Rakai Hospitals in Kyotera and Rakai districts. Data were collected from 15th December 2020 and 14th January 2021. Data were analyzed using a thematic content approach with the help of NVivo.<br><br>Results<br><br>Of the 20 key informants, 13 were females. In terms of work duration, 11 had worked with people living with HIV for 11-15 years and 9 were nurses. The challenges in managing HIV patients with NCDs included difficulty managing adverse events, heavy workload, inadequate communication from specialists to lower cadre health workers especially nurses and clinical officers following referrals, limited financial and human resources, unsupportive clinical guidelines that do not incorporate non-communicable disease management in HIV care and treatment and inadequate knowledge and skills required to appropriately manage non-communicable diseases. Health workers suggested integrating non-communicable disease management into routine HIV care and requested training before this integration.<br><br>Conclusion<br><br>Integration of non-communicable disease management into routine HIV care might help to reduce the burden of managing non-communicable diseases among health workers. Furthermore, training of health workers is required prior to integration.
Order of Authors:	Asani Kasango, MPH
	Alex Daama

Powered by Editorial Manager® and ProduXion Manager® from Aries Systems Corporation

	Lilian Negesa
**Additional Information:**	
**Question**	**Response**
**Financial Disclosure**<br><br>Enter a financial disclosure statement that describes the sources of funding for the work included in this submission. Review the submission guidelines for detailed requirements. View published research articles from *PLOS ONE* for specific examples.<br><br>This statement is required for submission and **will appear in the published article** if the submission is accepted. Please make sure it is accurate.<br><br>**Funded studies**<br>Enter a statement with the following details:<br>• Initials of the authors who received each award<br>• Grant numbers awarded to each author<br>• The full name of each funder<br>• URL of each funder website<br>• Did the sponsors or funders play any role in the study design, data collection and analysis, decision to publish, or preparation of the manuscript?<br><br>Did you receive funding for this work?	The author(s) received no specific funding for this work.
**Competing Interests**<br><br>Use the instructions below to enter a competing interest statement for this submission. On behalf of all authors, disclose any competing interests that could be perceived to bias this work—acknowledging all financial support and any other relevant financial or non-financial competing interests.<br><br>This statement is required for submission and **will appear in the published article** if the submission is accepted. Please make sure it is accurate and that any funding	The authors have declared that no competing interests exist.

sources listed in your Funding Information later in the submission form are also declared in your Financial Disclosure statement.<br><br>View published research articles from *PLOS ONE* for specific examples.<br><br>**NO authors have competing interests**<br><br>Enter: *The authors have declared that no competing interests exist.*<br><br>**Authors with competing interests**<br><br>Enter competing interest details beginning with this statement:<br><br>*I have read the journal's policy and the authors of this manuscript have the following competing interests: [insert competing interests here]*<br><br>* typeset	
**Ethics Statement**<br><br>Enter an ethics statement for this submission. This statement is required if the study involved:<br><br>• Human participants<br>• Human specimens or tissue<br>• Vertebrate animals or cephalopods<br>• Vertebrate embryos or tissues<br>• Field research<br><br>Write "N/A" if the submission does not require an ethics statement.<br><br>General guidance is provided below. Consult the submission guidelines for detailed instructions. **Make sure that all information entered here is included in the Methods section of the manuscript.**	Ethical review and approval were obtained from Makerere University School of Public Health Higher Degrees Research and Ethics Committee (HDREC) prior to data collection.<br>Administrative clearances were also sought from Kyotera District Health Officer (DHO) and Kalisizo Hospital Director prior to conducting Key informant interviews.<br>Written informed consents were obtained from respondents prior to participation in the study. These consents were written and administered in either Luganda or English.<br>All the collected data was stored with utmost confidentiality using password protected computers, only accessible to the study team.

**Format for specific study types**<br><br>**Human Subject Research (involving human participants and/or tissue)**<br>• Give the name of the institutional review board or ethics committee that approved the study<br>• Include the approval number and/or a statement indicating approval of this research<br>• Indicate the form of consent obtained (written/oral) or the reason that consent was not obtained (e.g. the data were analyzed anonymously)<br><br>**Animal Research (involving vertebrate animals, embryos or tissues)**<br>• Provide the name of the Institutional Animal Care and Use Committee (IACUC) or other relevant ethics board that reviewed the study protocol, and indicate whether they approved this research or granted a formal waiver of ethical approval<br>• Include an approval number if one was obtained<br>• If the study involved *non-human primates*, add *additional details* about animal welfare and steps taken to ameliorate suffering<br>• If anesthesia, euthanasia, or any kind of animal sacrifice is part of the study, include briefly which substances and/or methods were applied<br><br>**Field Research**<br><br>Include the following details if this study involves the collection of plant, animal, or other materials from a natural setting:<br>• Field permit number<br>• Name of the institution or relevant body that granted permission	
**Data Availability**<br><br>Authors are required to make all data underlying the findings described fully available, without restriction, and from the time of publication. PLOS allows rare exceptions to address legal and ethical concerns. See the PLOS Data Policy and FAQ for detailed information.	No - some restrictions will apply

Powered by Editorial Manager® and ProduXion Manager® from Aries Systems Corporation

A Data Availability Statement describing where the data can be found is required at submission. Your answers to this question constitute the Data Availability Statement and **will be published in the article**, if accepted.<br><br>**Important:** Stating 'data available on request from the author' is not sufficient. If your data are only available upon request, select 'No' for the first question and explain your exceptional situation in the text box.<br><br>Do the authors confirm that all data underlying the findings described in their manuscript are fully available without restriction?	
**Describe where the data may be found in full sentences. If you are copying our sample text, replace any instances of XXX with the appropriate details.**<br><br>• If the data are **held or will be held in a public repository**, include URLs, accession numbers or DOIs. If this information will only be available after acceptance, indicate this by ticking the box below. For example: *All XXX files are available from the XXX database (accession number(s) XXX, XXX.*).<br>• If the data are all contained **within the manuscript and/or Supporting Information files**, enter the following: *All relevant data are within the manuscript and its Supporting Information files.*<br>• If neither of these applies but you are able to provide **details of access elsewhere**, with or without limitations, please do so. For example:<br><br>*Data cannot be shared publicly because of [XXX]. Data are available from the XXX Institutional Data Access / Ethics Committee (contact via XXX) for researchers who meet the criteria for access to confidential data.*<br><br>*The data underlying the results presented in the study are available from (include the name of the third party*	The datasets used and/or analyzed during the current study are available from the corresponding author on reasonable request.

and contact information or URL).<br>• This text is appropriate if the data are owned by a third party and authors do not have permission to share the data.<br><br>* typeset	
Additional data availability information:	Tick here if the URLs/accession numbers/DOIs will be available only after acceptance of the manuscript for publication so that we can ensure their inclusion before publication.; Tick here if your circumstances are not covered by the questions above and you need the journal’s help to make your data available.

Manuscript

Click here to access/download;Manuscript;manuscript plos one Asani Kasango.docx

Challenges in managing Persons living with HIV w[illegible] non-communicable diseases and the perception of health workers towards integrated management of non-communicable diseases in routine HIV care in South Central Uganda: A qualitative study.

Asani Kasango[1,] Alex Daama[1,2] Lilian Negesa[1]

[1] Rakai Health Sciences Program

[2] African Medical and Behavioral Sciences Organization

Corresponding author: Asani Kasango

Kalisizo, Masaka City, Uganda.

Phone number: +256757980414/+256787165300

Hassankasango25@gmail.com

ABSTRACT

Background: Non-communicable diseases have been reported to be common among adults living with HIV. However, data on health systems challenges in managing non-communicable diseases and the perception of health workers towards integrated management of non-communicable diseases in routine care are limited in rural Ugandan settings.

This study therefore explored health system challenges in managing PLHIV with non-communicable diseases and health workers' perception regarding integration of non-communicable diseases in routine HIV care in South Central Uganda.

Methods: In this qualitative study, we collected data from 20 purposively selected key informants from Kalisizo Hospital and Rakai Hospital in South Central Uganda. Data were collected from 15th December 2020 and 14th January 2021. Data were analyzed using a thematic content approach with the help of NVivo.

Results: Of the 20 health workers, 13 were females. In terms of work duration, 11 had worked with people living with HIV for 11-15 years and 9 were nurses. The challenges in managing HIV patients with non-communicable diseases included difficulty managing adverse events, heavy workload, inadequate communication from specialists to lower cadre health workers especially nurses and clinical officers following referrals, limited financial and human resources, unsupportive clinical guidelines that do not incorporate non-communicable disease management in HIV care and treatment and inadequate knowledge and skills required to appropriately manage non-communicable diseases. Health workers suggested integrating non-communicable disease management into routine HIV care and suggested the need for training before this integration.

Conclusion: Integration of non-communicable disease management into routine HIV care might help to reduce the burden of managing non-communicable diseases among health workers. Furthermore, training of health workers is required prior to integration.

Introduction

It is estimated that over 70 million people have been infected with HIV. Furthermore, over 30 million people have died from acquired immunodeficiency syndrome (AIDS) since the 1980s [1]. Sub-Saharan Africa contributes to 90% of all people living with HIV (PLHIV) [2]. The wide scale-up of antiretroviral therapy (ART) has reduced the impact of the HIV epidemic. However, it has contributed to the non-

communicable diseases (NCDs) burden among PLHIV[3]. Non-communicable diseases place a huge burden on the healthcare system as they increase healthcare costs [4], [5] and overburden the health workers with the need to manage NCD independent of HIV. Furthermore, they require [illegible]t of consultations and unplanned hospital admissions [6]–[8].

About 6% of Ugandan adults live with HIV [9]. Of these, 20% have an NCD [10]. Among those with an NCD, adherence to NCD treatment is less than 30% [11], [12]. Therefore, integration of NCD management in routine HIV care and treatment could offer an opportunity to improve adherence to treatment. It could further reduce the burden on the health workforce [13].

In Uganda, little is known about the health systems challenges in managing HIV patients with NCDs. Furthermore, little is known about the perceptions of health workers towards integration of non-communicable disease management in routine HIV care. This study therefore explored the health systems challenges in managing NCDs among PLHIV. It also explored the perception of health workers towards NCD integration in routine care in south Central Uganda.

METHODS

Study design: We conducted a qualitative exploratory study utilizing key informant interviews with health workers.

Setting and population: Kalisizo Hospital is a Ministry of Health (MoH) Hospital located in Kalisizo town, Kyotera district. The Hospital is approximately 30kms from Masaka Regional Referral Hospital. The Hospital provides outpatient and inpatient services including maternal, reproductive, child health, nutritional, HIV, NCD screening and management and general health care. Kalisizo Hospital is the main Hospital for Kyotera district and serves as the referral Hospital for lower-level health facilities within the district. It has a catchment population of approximately 70,000 people [14]–[16]. The Hospital has a bed capacity of 120 beds. Over 87% of the population served live in rural areas who majorly depend on agriculture, trading, and fishing. HIV prevalence in Kyotera district is greater than 10% [17].

Rakai Hospital is a MOH Hospital located in the town of Rakai, Rakai district, about 61 kms southwest of Masaka Regional referral Hospital. It is the M[illegible]in and only Hospital in the district, and it provides in-patient and outpatient services including maternal, reproductive, child health, nutrition, HIV, NCD screening and management and general Healthcare. It is a predominantly a rural facility with a bed capacity of 100 beds [18]. Included in the study were health workers who were either working at Kalisizo

or Rakai Hospital, had 1 or more years of experience managing PLHIV and consented to participate in the study.

Sample and recruitment : The sample size for the study was guided by saturation principle and 20 key informant interviews were done. Of the key informants, 12 were selected from Kalisizo Hospital and 8 were selected from Rakai Hospital. Purposive sampling was used to select key informants.

Data collection:

Data were collected from 15th December 2020 and 14th January 2021 using a key informant interview guide with standardized open-ended questions. The tool contained questions about the common NCDs among PLHIV, questions on how they are managed, the challenges in their management, what could improve their management and what they thought about integrated management of NCDs in routine HIV care. With the key informant's permission, field notes were taken and were supplemented with audio recordings. All the key informant interviews were conducted in English and lasted 40 to 60 minutes.

Data Analysis.

Data were managed using NVivo software. The thematic content analysis approach, as proposed by Bardin in 1977, was adopted to analyze data. Operationally, all the significant statements in the interviews were open coded (that is, we did not use pre-set codes), and then some clusters of codes were identified as themes. Then, these themes were circumscribed into thematic categories, forming analogical groups, classified, and aggregated according to what was understood about their meanings for the study participants. The interviews were analyzed and coded one by one after carefully reading the transcripts and field notes. This analysis led to saturation of the results, as we had sufficient elements to reach the proposed objective. Finally, the thematic categories and respective themes were examined and discussed with the aim of adopting a comprehensive and interpretive approach of the results.

RESULTS

Characteristics of Key informants

Of the 20 key informants, 13 were females. In terms of work duration, 11 had worked with PLHIV for 11-15 years and 9 were nurses. This is presented in table 1.

Table 1: Characteristics of key informants.

KI#	SEX	CADRE	PERIOD WORKING WITH PLHIV (Years)
01	M	Counselor	14
02	F	Nurse	15
03	F	Nurse	6
04	M	Doctor	1
05	F	Nurse	12
06	F	Nurse	14
07	F	Clinical Officer	6
08	M	Clinical Officer	7
09	M	Clinical Officer	8
10	F	Nurse	13
11	F	Nurse	5
12	F	Clinical officer	9
13	M	Public Health	5
14	F	PUBLIC HEALTH	7
15	F	NURSE	11
16	F	NURSE	10
17	F	CLINICAL OFFICER	11
18	F	NURSE	5
19	M	Counselor	14
20	M	Doctor	11

The common NCDs among people living with HIV:

Metabolic and cardiovascular diseases especially diabetes and hypertension were reported to be common among PLHIV.

"Metabolic conditions are commonly reported among PLWHIV on care…… Diabetes is less common as *compared to hypertension……these conditions are common among PLWHIV compared to those who are HIV negative".***(KI09).**

"When you take blood pressures say for 10 people, two to three will have high blood pressure" **(KI 02).**

"*During counseling sessions, a lot of clients express concerns that they are experiencing blood pressure like symptoms….when their blood pressures are measured, most are told that they are hypertensive…."(***KI 19).**

Mental health conditions were also reported to be common among PLHIV at Kalisizo Hospital, prominent among others was depression.

"In addition to other mental health conditions, depression is common among our patients.....One of our client was severely depressed after being diagnosed with diabetes and hypertension.... she attempted suicide twice, though we were able to offer her psychotherapy and thank God, she's still alive.......many more have depression" **(KI 11).**

"Depression is like a sister to HIV at the hospital....Many will become worried to the extent of failing to perform normal duties and on investigation, a diagnosis comes positive for depression" **(KI 20).**

"Depression is very common here...one in every three clients will experience depressive symptoms at least one a year" **(KI 01).**

"Generally, mental health concerns are highly prevalent here...We even have clients who have ever attempted suicide..." **(KI 15).**

Health systems challenges in managing HIV patients with NCDs:

Polypharmacy:

Although health workers perceived that most of their patients had low viremia, they noted challenges in coordinating medications, managing adverse events, handling unwanted drug interactions when coordinating HIV management with treatments for NCDs. Dealing with multiple medications and medication interactions were common concerns of health workers:

"People living with HIV who have NCDs come to the hospital feeling sad due to multiple medications... Some will even tell you they are done with certain drugs and others say they are tired of taking all drugs...we sometimes become confused how well to handle interaction given that you prescribe these drugs knowing possibilities of interaction and adverse events". **(KI 08).**

"A patient will come with drug related complaints, and you may end up giving them all your attention and ignoring your other patients....managing drug interaction and adverse event is burdensome............". **(KI 02).**

"Polypharmacy has always challenged us....I don't know if drug manufacturers could think of supplying drugs that can treat multiple non-communicable diseases...this could help to reduce our major hurdle: Polypharmacy" **(KI 20).**

Poor communication and limited support from highly specialized physicians:

Additionally, health workers had concerns arising from referrals to other health care providers who do not maintain effective communication and feedback with them. One KI said,

"We sometimes refer patients to specialized physicians who sometimes don't communicate effectively with us. They'll keep quiet and only communicate back to us occasionally". **(KI 06).**

"*The specialists communicate to us occasionally whenever we refer patients, and we are sometimes forced to wait for their communication before offering care and treatment to these patients and this makes the treatment process difficult…."* **(KI 09).**

Inadequate resources to manage NCDs:

Inadequate resources also challenge NCD management among PLHIV at the Hospital. Managing NCDs is difficult for practitioners especially when compounded with socioeconomic deprivation and limited budgetary allocation. Managing NCDs is financially costly yet the budget allocated to care, and treatment of these conditions is limited. One KI noted:

"Some of these conditions especially hypertension and type 2 diabetes may require primary and secondary care, outpatient visits, and hospital admissions and these strain the Hospital's limited resources". **(KI 20).**

Another KI recounted:

"Each of these conditions is expensive to manage independently and you can imagine if an individual has multiple conditions". **(KI 04).**

Another KI stated that:

"When compared to their HIV negative counterparts, PLHIV suffer from condition which weaken their immunity and makes them vulnerable to more infections. Some of these conditions require out of pocket expenditure, yet most of these persons are in dire economic state making it difficult to buy medicines that they require to treat these conditions……they are required to feed well to stay healthy. However, some of these individuals do not have money to buy food required to maintain healthy diet prior to medication and

they sometimes come to the facility seeking some support and we are sometimes required to give financial and non-financial support in terms of food making it difficult for us and the patients". **(KI 09).**

Unsupportive clinical guidelines:

The clinical guidelines focus on management of single conditions, and this hinders NCD management in this vulnerable population. One KI said:

"It may be easier for us to manage HIV alone. However, if physical and mental health conditions are present, we are unable to consult the clinical guidelines on the proper management of these conditions and this affects our work ....". **(KI 07).**

Another KI said:

"…You can only consult the available clinical guidelines if your patient has a single condition…when managing multiple conditions, the available clinical guidelines become less supportive…" **(KI 13).**

Limited knowledge and training in NCD management:

Lack of specialized knowledge, training and expertise also hinders NCD management at the Kalisizo Hospital. One of the KIs said:

"Our training both in class and through Continuous medical education makes us competent in managing single conditions. Managing NCDs alongside HIV is challenging to most of us because it is not part of our training….most of staff are clinical officers and nurses and are trained to manage single conditions……even when we check our clinical guidelines, they focus on single conditions……". **(KI 12).**

" *A few of us here can manage multiple conditions simultaneously and for most, we feel we have a lot of knowledge gaps….".* **(KI 18).**

Perceptions towards integrated management of NCDs in routine care:

Of the 20 key informants, most (fifteen) recommended integrated management of NCDs in routine care.

"Personally, I think by integrating non-communicable diseases in routine HIV care, it will reduce our workload….we will have more time and we will be able to give our clients the kind of care they deserve, and this will improve non-communicable disease management…" **(KI 20).**

"*By managing multiple conditions simultaneously, it saves time, costs and the burden on our side".* **(KI 17).**

"Although it is important to integrate NCDs in routine care, a lot of efforts are desired including providing us with continuous medical education and training, increasing the resource base…." **(KI 20).**

A few who did not recommend integrated management had several concerns including budgetary allocation, additional time it will require, and the technical knowledge required. For instance, a KI asserted:

"I don't think integration is what we need. Our budgetary allocation and human resource capacity cannot facilitate it and I don't think we are ready for it yet…" **(KI 14)**.

"I am not knowledgeable enough about managing non-communicable diseases and I don't recommend integration" **(KI 02).**

DISCUSSION

We explored the common non-communicable diseases among PLHIV, the health systems challenges in managing NCDs and the perception of health workers towards integrated management of NCDs during routine HIV care at Kalisizo and Rakai Hospital. Hypertension and mental health conditions especially depression were commonly treated by health workers. The challenges that health workers experienced include: polypharmacy, inadequate communication from specialists to lower cadre health workers especially nurses and clinical officers following referral, limited financial and human resources, unsupportive clinical guidelines which did not incorporate non-communicable disease management in HIV care, and treatment and inadequate knowledge and skills required to appropriately manage non-communicable diseases. Majority of the health workers recommended integration of NCDs in routine HIV care.

In our study, hypertension and mental health concerns were commonly reported by PLHIV who were receiving care at the Hospitals. This is consistent with findings from [19], [20]. The possible explanation

for the consistency could be because HIV increases self and public stigma which makes PLHIV vulnerable to mental health concerns [21].

Furthermore, health workers reported several challenges in managing HIV patients with NCDs. For instance, polypharmacy and adverse events are common among NCD patients [22], [23]. Multiple drug therapy in multiple disease management is a common area of difficulty for both patients and physicians [24], [25]. Polypharmacy is common in multiple disease management because guidelines are single disease focused and advise when to start new drugs but seldom when to stop them. The more LTCs (long term conditions) a patient has the more medications they are likely to be prescribed [25]. Polypharmacy commonly leads to drug disease and drug-drug interactions [26].

Limited communication from higher level officers to lower-level health workers also emerged as a key theme from our results, and this is consistent with findings from previous studies. For instance, a study on multimorbidity reported that the involvement of several medical specialists, who each emphasize the importance of 'their' 'guideline' and the 'poor communication from specialists and hospitals to the family physician' made coordination and medication hard to maintain. Despite these reservations, the lower cadre workers desired input of specialist [26]. Our findings also reported unsupportive clinical guidelines, inadequate resources and inadequate knowledge and skills in incorporating NCD management in routine HIV care as key challenges in managing HIV patients with NCDs and these results are consistent with findings from [6], [7], [27].

Most health workers preferred integrated management of NCDs in routine care and this is consistent with quantitative results from [28]. Furthermore, a qualitative study done in Tanzania reported that both health workers and clients preferred integrated management of HIV and NCDs [29].

This study has several strengths for example, this study was conducted by qualified and experienced research assistants with high expertise in data collection and probing skills and this significantly improved the quality data collected. The study was conducted at two facilities and this increased generalizability of findings.

Study Limitations:

This study did not interview District Health officials. We therefore recommend studies involving district health staff.

Conclusions

Integrating NCD management in routine HIV care and designing supportive clinical guidelines coupled with timely, focused, and adequate communication from higher to lower-level health workers are critical for proper management of HIV among PLHIV.

Declaration

Ethical considerations

Ethical review and approval were obtained from Makerere University School of Public Health Higher Degrees Research and Ethics Committee (HDREC) prior to data collection.

Administrative clearances were also sought from Kyotera District Health Officer (DHO) and Kalisizo Hospital Director prior to conducting Key informant interviews.

Written informed consents were obtained from respondents prior to participation in the study.

All the collected data was stored with utmost confidentiality using password protected computers, only accessible to the study team.

Availability of data and materials:

The datasets used and/or analyzed during the current study are available from the corresponding author upon reasonable request.

Consent to publish.

Not applicable.

Competing interests [Mandatory]

We declare no conflict of interest.

Acknowledgements [Mandatory]

We would like to thank Kalisizo Hospital and Rakai Hospital for hosting this study and in the same spirit thank our participants for their great participation and making this study real.

Funding:

The study was privately funded.

Authors' contributions [Mandatory]

Asani Kasango MPH contributed to the conception of this research idea, study design, data analysis and including supporting all stages of this paper. Mr. Alex Daama MPH supported with the study design, study conceptualization, data analysis, and interpretation of findings. All authors reviewed the study.

Disclaimer:

The findings and conclusions in this report are those of the author(s) and do not necessarily represent the official position of Rakai Health Sciences Program and African Medical and Behavioral Sciences Organization.

References

[1] J. S. Lozada, "Joint United Nations Programme on HIV/AIDS," *Encycl. Glob. Heal.*, 2012, doi: 10.4135/9781412963855.n665.

[2] P. Patel *et al.*, "Noncommunicable diseases among HIV-infected persons in low- income and middle-income countries: a systematic review and meta-analysis," *HHS Public Access*, vol. 32, no. Suppl 1, pp. 1–28, 2019, doi: 10.1097/QAD.0000000000001888.Noncommunicable.

[3] K. M. Venkat Narayan *et al.*, "HIV and noncommunicable disease comorbidities in the era of antiretroviral therapy: A vital agenda for research in low-and middle-income country settings," *J. Acquir. Immune Defic. Syndr.*, vol. 67, no. SUPPL.1, pp. 2–7, 2014, doi: 10.1097/QAI.0000000000000267.

[4] A. Prados-Torres, A. Calderón-Larrañaga, J. Hancco-Saavedra, B. Poblador-Plou, and M. Van Den Akker, "Multimorbidity patterns: A systematic review," *J. Clin. Epidemiol.*, vol. 67, no. 3, pp. 254–266, 2014, doi: 10.1016/j.jclinepi.2013.09.021.

[5] I. M. Magodoro, T. M. Esterhuizen, and T. Chivese, "A cross-sectional, facility based study of comorbid non-communicable diseases among adults living with HIV infection in Zimbabwe," *BMC Res. Notes*, vol. 9, no. 1, pp. 1–10, 2016, doi: 10.1186/s13104-016-2187-z.

[6] M. Moffat, "Challenges of managing people with multimorbidity in today's healthcare systems.," *BMC Fam Pr.*, 2015, doi: 10.1186/s12875-015-0344-4.

[7] J. Slomka, M. Prince-Paul, A. Webel, and B. J. Daly, "Multimorbidity With HIV: Views of Community-Based People Living With HIV and Other Chronic Conditions," *J. Assoc. Nurses*

AIDS Care, vol. 28, no. 4, pp. 603–611, 2017, doi: 10.1016/j.jana.2017.04.003.

[8] J. Kiplagat, A. Mwangi, C. Chasela, and S. Huschke, "Challenges with seeking HIV care services: Perspectives of older adults infected with HIV in western Kenya," *BMC Public Health*, vol. 19, no. 1, pp. 1–12, 2019, doi: 10.1186/s12889-019-7283-2.

[9] N. et al. Musoba, "Uganda HIV / AIDS Country Progress Report July 2016-June 2017," *Uganda AIDS Com.*, no. JUNE, 2017.

[10] S. Kansiime, D. Mwesigire, and H. Mugerwa, "Prevalence of non-communicable diseases among HIV positive patients on antiretroviral therapy at joint clinical research centre, Lubowa, Uganda," *PLoS One*, vol. 14, no. 8, pp. 1–11, 2019, doi: 10.1371/journal.pone.0221022.

[11] M. Muddu *et al.*, "Treatment Program in Eastern Uganda : a retrospective cohort study," vol. 81, no. 5, pp. 552–561, 2020, doi: 10.1097/QAI.0000000000002067.Integrated.

[12] D. Sando, A. Kintu, S. Okello, P. C. Kawungezi, D. Guwatudde, and G. Mutungi, "Cost-effectiveness analysis of integrating screening and treatment of selected non-communicable diseases into HIV / AIDS treatment in Uganda," vol. 23, 2020, doi: 10.1002/jia2.25507.

[13] D. Bukenya *et al.*, "PLOS GLOBAL PUBLIC HEALTH Integrated healthcare services for HIV , diabetes mellitus and hypertension in selected health facilities in Kampala and Wakiso districts , Uganda : A qualitative methods study," pp. 1–23, 2022, doi: 10.1371/journal.pgph.0000084.

[14] V. L. Kiggundu *et al.*, "High prevalence of malaria parasitemia and anemia among hospitalized children in Rakai, Uganda," *PLoS One*, vol. 8, no. 12, pp. 8–13, 2013, doi: 10.1371/journal.pone.0082455.

[15] Mayuge district, "Mayuge district Performance Report." MK, kampala, 2019.

[16] A. Zubairu, Iliyasu; Aminatu A, Kwaku; Amina A, Umar; Fatimah, Tsiga-Ahmed; Nafisa S, Nass; Hadiza M, Abdullahi; Taiwo G., Amole; Hamisu M., Salihu; Muktar H., "Predictors of COVID-19 Vaccine Acceptability among Patients Living with HIV in Northern Nigeria: A Mixed Methods Study," *Minist. Heal. Uganda*, no. November, pp. 1–164, 2022, doi: 10.2174/1570162X19666211217093223.

[17] L. W. Chang *et al.*, "Heterogeneity of the HIV epidemic in agrarian, trading, and fishing communities in Rakai, Uganda: an observational epidemiological study," *Lancet HIV*, vol. 3, no. 8, pp. e388–e396, 2016, doi: 10.1016/S2352-3018(16)30034-0.

[18] Ministryof Health Uganda, "Rakai Hospital," 2016.

[19] R. Rutakumwa *et al.*, "Research Article A Qualitative Exploration of Causes of Depression

among Persons Living with HIV Receiving Antiretroviral Therapy in Uganda : Implications for Policy,” vol. 2023, 2023.

[20] Y. Damtie, B. Kefale, M. Yalew, and M. Arefaynie, “Depressive symptoms and associated factors among HIV positive patients attending public health facilities of Dessie town : A cross-sectional study,” *PLoS One*, pp. 1–12, 2021, doi: 10.1371/journal.pone.0255824.

[21] H. Atwijukiire *et al.*, “Stigma and Discrimination of HIV / AIDs Recipients of Care in Western Uganda : Experiences and Roles of Expert Clients,” no. November, pp. 517–527, 2022.

[22] E. Søndergaard *et al.*, “Problems and challenges in relation to the treatment of patients with multimorbidity : General practitioners ’ views and attitudes *,” no. February, pp. 121–126, 2015, doi: 10.3109/02813432.2015.1041828.

[23] S. M. Smith, H. Soubhi, M. Fortin, and C. Hudon, “Managing patients with multimorbidity : systematic review of interventions in primary care and community,” vol. 5205, no. September, pp. 1–10, 2012, doi: 10.1136/bmj.e5205.

[24] K. Payne, “Adapting clinical guidelines to take account of multimorbidity Deposited on : 19 October 2012 Enlighten – Research publications by members of the University of Glasgow,” no. March 2014, pp. 2009–2013, 2012, doi: 10.1136/bmj.e6341.

[25] C. Sinnott, S. M. Hugh, J. Browne, and C. Bradley, “GPs ’ perspectives on the management of patients with multimorbidity : systematic review and synthesis of qualitative research,” 2013, doi: 10.1136/bmjopen-2013-003610.

[26] A. W. Taylor *et al.*, “Multimorbidity - not just an older person ’ s issue . Results from an Australian biomedical study,” pp. 16–18, 2010.

[27] S. M. McPhail, “Multimorbidity in chronic disease: Impact on health care resources and costs,” *Risk Manag. Healthc. Policy*, vol. 9, pp. 143–156, 2016, doi: 10.2147/RMHP.S97248.

[28] T. Masupe, Y. Mashalla, E. Seloilwe, H. Jibril, and H. Medhin, “Integrated management of HIV / NCDs : knowledge , attitudes , and practices of health care workers in Gaborone , Botswana,” *Afr. Health Sci.*, vol. 19, no. 3, pp. 2312–2323, 2019.

[29] E. H. Shayo *et al.*, “The acceptability of integrated healthcare services for HIV and non‑communicable diseases : experiences from patients and healthcare workers in Tanzania,” *BMC Health Serv. Res.*, pp. 1–12, 2022, doi: 10.1186/s12913-022-08065-4.
