# Supplementary material for: Challenges in managing HIV and non-communicable diseases and health workers’ perception regarding integrated management of non-communicable diseases during routine HIV care in South Central Uganda: A qualitative study
Source: PLoS One. 2024 Aug 20;19(8):e0302290. doi: 10.1371/journal.pone.0302290 (PMC11335126; doi:10.1371/journal.pone.0302290)
Supplement: Attachment — Submitted filename: Response to Reviewers.docx [file pone.0302290.s004.docx]

**POINT-BY-POINT REBUTTAL LETTER**

We are grateful for the opportunity to revise our manuscript titled “**Challenges in managing HIV and non-communicable diseases and health workers’ perception regarding integrated management of non-communicable diseases during routine HIV care in South Central Uganda: A qualitative study**”. In response to the insightful feedback from the editor and reviewers, we have revised the manuscript, incorporating changes and additions where necessary. Below, we provide a detailed response to each comment raised by the reviewer. The format of our responses is organized as follows: a) Comments from editors or reviewers are presented in standard text. b) Our responses to each comment are indicated directly beneath the comment and are highlighted in red for clarity.

**Journal Requirements**

When submitting your revision, we need you to address these additional requirements.

1. Please ensure that your manuscript meets PLOS ONE's style requirements, including those for file naming. The PLOS ONE style templates can be found at

https://journals.plos.org/plosone/s/file?id=wjVg/PLOSOne_formatting_sample_main_body.pdf and https://journals.plos.org/plosone/s/file?id=ba62/PLOSOne_formatting_sample_title_authors_ affiliations.pdf

***We have checked the templates and made the necessary changes, including file naming. we have also formatted the manuscript to reflect the PLOS One formatting style.***

2. Note from Emily Chenette, Editor in Chief of PLOS ONE, and Iain Hrynaszkiewicz, Director of Open Research Solutions at PLOS: Did you know that depositing data in a repository is associated with up to a 25% citation advantage (https://doi.org/10.1371/journal.pone.0230416)? If you’ve not already done so, consider depositing your raw data in a repository to ensure your work is read, appreciated and cited by the largest possible audience. You’ll also earn an Accessible Data icon on your published paper if you deposit your data in any participating repository (https://plos.org/open-science/open-data/#accessible-data)..

## ***All findings derived from the study are fully disclosed and published in the main manuscript body. The data underlying these findings has been published in Figshare.***

3. In the online submission form, you indicated that "The datasets used and/or analyzed during the current study are available from the corresponding author on reasonable request."
    
   All PLOS journals now require all data underlying the findings described in their manuscript to be freely available to other researchers, either 1. In a public repository, 2. Within the manuscript itself, or 3. Uploaded as supplementary information.
   This policy applies to all data except where public deposition would breach compliance with the protocol approved by your research ethics board. If your data cannot be made publicly available for ethical or legal reasons (e.g., public availability would compromise patient privacy), please explain your reasons on resubmission and your exemption request will be escalated for approval..

## ***All findings derived from the study are fully disclosed and published in the main manuscript body.***

Your ethics statement should only appear in the Methods section of your manuscript. If your ethics statement is written in any section besides the Methods, please move it to the Methods section and delete it from any other section. Please ensure that your ethics statement is included in your manuscript, as the ethics statement entered into the online submission form will not be published alongside your manuscript.

## ***The ethics statement has been moved the methods section of our manuscript.***

Please review your reference list to ensure that it is complete and correct. If you have cited papers that have been retracted, please include the rationale for doing so in the manuscript text, or remove these references and replace them with relevant current references. Any changes to the reference list should be mentioned in the rebuttal letter that accompanies your revised manuscript. If you need to cite a retracted article, indicate the article’s retracted status in the References list and also include a citation and full reference for the retraction notice.

***The current reference list is complete and correct. We removed retracted papers from the references.***
- Reviewer #2: The introduction is scanty and does not give adequate background. Enrichment with more literature references would be most welcome.

***We have revised our introduction and added more literature.***

Reference the statistical software used to analyse eg. Nvivo software. -

***We have referenced the statistical software used (Nvivo).***

Table 1: Cadre: Participants 14-18 are written in caps, change to small caps for consistency.

***We have changed Table 1 Cadre: Participants 14-18 to small caps to ensure consistency.***

Line 98 “ In terms of work duration, 11 had worked with PLHIV for 11-15 years and 9 were nurses” However, in Table 1: only 9 participants had worked for 11-15 years and 6 were nurses.

***We have made the necessary changes as follows:*** **Of the 20 key informants, 13 were females. In terms of work duration, 9 had worked with PLHIV for 11-15 years and 9 were nurses**.

There is no consistency with writing abbreviations. Eg: PLHIV vs PLWHIV

***We have made our abbreviations consistent and maintained PLHIV.***

Under ethics statement they mentioned that the consents were administered in either Luganda or English, however, in line 85 they mentioned that interviews were conducted in English.

***We administered our consent in English, and that was a typo.***

The statistical analysis is not in deep described and is not clear which statistical analysis was performed for descriptive analysis.

***We have revised the data analysis section to reflect statistical analysis.***

Under the method section, clarity on how the participant number was calculated to achieve satisfactory statistical power for appropriate interpretation of results is needed.

***We were guided by the saturation principle, and we reached saturation at the 20^th^ interview.***

The discussion section should be deep reorganized. Discussion section should relates to the literature review and research questions, and making an argument in support of your overall conclusion.

***We have reorganized the discussion section and it now relate well with literature review, research questions and supports the overall conclusion.***

Does this refer to efforts to escalate care for those in need - which doesnt come out clearly in this paper. It would help to clarify 1. could health care workers escalate their patients as needed into advanced tiers of care 2. was the poor health communication around efforts to escalate care or feedback from practitioners to whom care was escalated

***This was addressed in the manuscript body.***
